# Sex differences in the physical inactivity and health-related quality of life relationship among rural adults

**DOI:** 10.15171/hpp.2016.30

**Published:** 2016-10-01

**Authors:** Peter D Hart

**Affiliations:** ^1^Health Promotion, Montana State University - Northern, Havre, MT, USA; ^2^Research and Statistical Consultant, Health Demographics, Havre, MT, USA

**Keywords:** Physical inactivity, Health-related quality of life (HRQOL), Rural health, Health disparities, Population health

## Abstract

**Background: ** The purpose of this study was to investigate the relationship between physical inactivity (PIA) and health-related quality of life (HRQOL) in rural adults and examine the extent to which sex differences exist in this relationship.

**Methods:** A total of 5617 adults 18 years of age and older who indicated residing in a rural county was included in this analysis. PIA status was assessed by questions regarding recreational physical activity during the previous month. Five HRQOL measures (physical health, mental health, inactivity health, general health, & unhealthy days) were used as primary outcome variables. PIA and HRQOL prevalence estimates were computed with 95% CIs. Multiple logistic regression was used to obtain odds ratios (ORs) and 95% CIs adjusted for age, ethnicity, and income.

**Results:** Physically inactive rural adults were significantly more likely to report poor HRQOL in all overall crude models with ORs ranging from 1.59 to 2.16. Additionally, sex-by-PIA interactions were significant across all crude HRQOL models with ORs ranging from 2.27 to 3.08 and 1.56 to 2.42 for women and men, respectively. Sex differences were maintained in fully adjusted models, except for mental health and inactivity health with ORs ranging from 1.80 to 2.58 and 1.41 to 1.79 for women and men, respectively.

**Conclusion:** Results from this study show that PIA is a strong predictor of poor HRQOL even after controlling for confounding variables. Furthermore, physically inactive rural women appear more likely to report poor levels of HRQOL than physically inactive rural men.

## Introduction

Physical inactivity (PIA) is a health risk behavior related to premature morbidity and mortality and has been of major public health focus since the landmark 1996 report entitled *Physical activity and health: a report of the Surgeon General*.^1^ Health-related quality of life (HRQOL) is an outcome measure of growing interest and has recently been added as a topic area to the decennial report by the United States Department of Health and Human Services (USDHHS) entitled *Healthy People 2020*.^2^ HRQOL is known to be superior among adults who participate in physical activity.^3^ In older adults, it has been shown that participating in no physical activity (PA) is related to lower reported HRQOL as compared to those who do participate in PA.^4^ Sedentary behavior (and regular PA) has also been associated with poorer HRQOL (and better HRQOL) in younger populations.^5^

Despite these known associations, inferences to rural populations are limited at best. Furthermore, there is also research that indicates adults residing in rural regions suffer disparities in HRQOL.^6^ As well, sex differences have been seen in both the PA^7,8^ and HRQOL^9,10^ literature, however, evidence of sex differences in the PA and HRQOL relationship is sparse. Therefore, the purpose of this study was to investigate the relationship between PIA and HRQOL in rural adults and examine the extent to which sex differences exist in this relationship.

## Materials and Methods

### Sample

Data for this study came from the 2013 Montana Behavioral Risk Factor Surveillance System (BRFSS). The BRFSS is administered by the Population Health Surveillance Branch of the Centers for Disease Control and Prevention (CDC) and was established in 1984.^11^ The annual survey is state-based (including territories) and comprises a cross-sectional sample of non-institutionalized US adults 18+ years of age. The survey is administered by telephone and collects responses to questions concerning health-related risk behaviors, health status, as well as participant use of preventive services. Rural status for inclusion was established from the metropolitan statistical area (MSA) variable which was part of the BRFSS dataset. Participants either assigned an MSA that had no center city or not assigned to an MSA were considered residing in a rural area. A total of 6103 adults who indicated residing in a rural area were initially used in the analysis.^12^

### Measures

PIA was assessed by responses to the following question: “During the past month, other than your regular job, did you participate in any physical activities or exercises such as running, calisthenics, golf, gardening, or walking for exercise”?^13^ Those respondents answering ‘no’ to this question were considered physically inactive and those answering ‘yes’ were considered not physically inactive. A continuous measure of self-reported body mass index (BMI) was recoded into BMI weight categories as follows: underweight (BMI <18.5 kg/m^2^), normal weight (BMI 18.5-24.9 kg/m^2^), overweight (BMI 25-29.9 kg/m^2^), and obese (BMI ≥ 30 kg/m^2^).^14^

The CDC’s Healthy Days core was used for the first four measures of HRQOL (general health, physical health, mental health, and inactivity health).^15^ The first measure came from a single question that asked about general health (general health). Those responding “fair” or “poor” were considered to have poor HRQOL and those responding “excellent”, “very good”, or “good” were considered to have good HRQOL. The next two measures came from questions that asked respondents how many days out of the previous 30 that their physical (physical health) and mental (mental health) health was not good. The fourth measure came from a question that asked respondents how many days from the previous 30 that their physical and/or mental health prevented them from doing their usual activities. A fifth *Healthy Days* index was computed representing the number of healthy days (unhealthy days) out of the previous 30. A cut-off of 14+ days was used to indicate ‘poor’ health for the previous four measures. These five measures were dichotomized to indicate poor HRQOL.^16^ Reliability of the CDC HRQOL items has been reported as moderate to excellent.^17^

### Statistical analysis

Prevalence estimates, standard errors (SEs), and Rao-Scott adjusted chi-square tests of independence were used to describe PA across demographic characteristics (Table 1). Prevalence estimates and crude odds ratios (ORs) with 95% CIs by logistic regression models were used to describe poor HRQOL by PIA status (Table 2). Multiple logistic regression models were used to calculate ORs and 95% CIs of reporting poor HRQOL among adults who reported no PA (Table 3), while adjusting for age, race, gender, and income.^18^ All analyses were performed using the complex samples module of SPSS version 24.^19,20^ All *P* values are reported as 2-sided and statistical significance was set at 0.05.

## Results

A total of 5617 rural adults had complete PA data to be included in the analysis (see Table 1). Overall, 27.8% of rural adults reported participating in no PA. Significant sex differences in the prevalence of PIA was seen with 29.9% of males reporting no PA as compared to 26.1% of females (*P *= 0.03). Examining PIA prevalence across demographic characteristics, no significant sex-differences were seen across race groups (*P *= 0.54) or income groups (*P *= 0.37). However, significant PIA differences were seen across age groups (*P *= 0.01), with a general pattern of females being less physically inactive than males.

Table 2 presents prevalence estimates and odds of poor HRQOL by PIA status. Among males, those who were physically inactive were significantly more likely to report poor physical health (22.1% vs. 12.2%, *P *< 0.001), mental health (12.2% vs. 5.4%, *P *< 0.001), inactivity health (28.2% vs. 16.9%, *P *= 0.004), general health (22.7% vs. 15.9%, *P *= 0.004), and unhealthy days (26.7% vs. 15.9%, *P *< 0.001). Females who were physically inactive showed even greater prevalence of poor HRQOL (all interaction *P* values**< 0.05). Specifically, those females who were physically inactive were significantly more likely to report poor physical health (26.8% vs. 10.6%, *P *< 0.001), mental health (17.2% vs. 8.4%, *P *< 0.001), inactivity health (25.4% vs. 12.4%, *P *< 0.001), general health (28.0% vs. 13.2%, *P *< 0.001), and unhealthy days (34.0% vs. 16.1%, *P *< 0.001).

Table 3 shows results for both age-adjusted and fully-adjusted analyses, modeling the odds of poor HRQOL among physically inactive (compared to physically active) rural adults. Sex-by-PIA interactions were significant (*P *< 0.05) across all fully-adjusted HRQOL models except mental health (overall combined OR: 2.12; 95% CI: 1.56-2.89) and inactivity health (overall combined OR: 1.95; 95% CI: 1.43-2.67). Physically inactive rural males were significantly more likely to report poor HRQOL in fully-adjusted models of physical health (OR: 1.79; 95% CI: 1.22-2.63), mental health (OR: 2.10; 95% CI: 1.28-3.44), inactivity health (OR: 1.92; 95% CI: 1.19-3.10), general health (OR: 1.41; 95% CI: 1.01-1.99), and unhealthy days (OR: 1.69; 95% CI: 1.18-2.42), compared to those males who were not physically inactive. Whereas, physically inactive rural females were significantly more likely to report poor HRQOL in fully-adjusted models of physical health (OR: 2.58; 95% CI: 1.86-3.59), mental health (OR: 2.16; 95% CI: 1.46-3.21), inactivity health (OR: 2.00; 95% CI: 1.31-3.05), general health (OR: 1.80; 95% CI: 1.32-2.47), and unhealthy days (OR: 2.43; 95% CI: 1.81-3.26), compared to those females who were not physically inactive.

**Figure 1:**
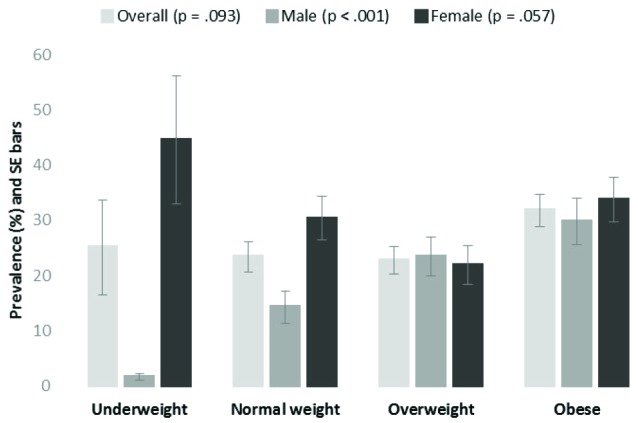
Prevalence of self-reported poor general health among physically inactive adults by body mass index (BMI) category, rural adults 2013.
Note: *P* values are for tests of linear trend in prevalence estimates across BMI category. BMI categories: underweight (BMI <18.5 kg/m^2^), normal weight (BMI 18.5-24.9 kg/m^2^), overweight (BMI 25-29.9 kg/m^2^), and obese (BMI ≥30 kg/m^2^).

Figure 1 graphs prevalence estimates of poor general health across BMI categories. Both underweight and normal weight females who were physically inactive were significantly (*P *< 0.05) more likely to report poor general health than their male counterparts. Sex differences in prevalence of poor general health were not evident in physically inactive overweight or obese. However, prevalence of poor general health was significantly (*P *< 0.05) greater in physically inactive obese females as compared to their overweight counterparts. Finally, the prevalence of poor general health followed a significant (*P *< 0.001) direct linear trend with BMI category for physically inactive males. Whereas poor general health prevalence followed a non-significant but suggestive (*P *= 0057) indirect linear trend with BMI category for physically inactive females.

**Table 1:** Prevalence of physical inactivity (PIA) by demographic characteristic, rural adults 2013

	**Physically inactive**						
	**Males**			**Females**			**Interaction**
**Characteristic **	**%**	**SE**	***P***	**%**	**SE**	***P***	***P***
Overall	29.9	1.4	<0.001	26.1	1.1	< 0.001	0.030
Age group (years)			0.254			<0.001	0.013
18-24	17.1	6.0		12.0	5.3		
25-34	31.6	5.2		15.5	2.9		
35-44	28.8	4.1		22.8	3.4		
45-54	34.2	3.6		25.1	2.9		
55-64	29.2	2.6		23.0	2.1		
65+	31.0	2.1		34.6	1.9		
Race/Ethnicity			0.493			0.176	0.540
White	29.7	1.5		26.0	1.2		
American Indian	30.6	3.9		32.5	3.4		
Hispanic	16.9	8.6		14.8	6.3		
Multiracial	42.1	10.4		26.6	12.0		
Other	41.8	21.0		12.9	9.0		
Income (US $)			0.001			< 0.001	0.370
<10 000	26.6	6.8		41.0	6.0		
10-14 999	39.2	5.7		33.7	4.6		
15-19 999	40.2	5.6		42.3	5.1		
20-24 999	38.6	4.4		28.2	3.3		
25-34 999	37.0	4.3		28.6	3.1		
35-49 999	31.4	3.3		24.0	2.8		
50-74 999	26.7	3.4		21.0	2.9		
75 000+	17.7	2.3		14.3	2.1		

Note: *P* values are for the Rao-Scott chi-square statistic or multiple logistic regression. Sex-specific *P* values are testing for differences in PIA status. Interaction *P* values are testing for sex-differences. Total sample size, N = 5617.

**Table 2:** Prevalence of poor HRQOL by PIA status, rural adults 2013

**Physically inactive**				
**Male **	**Yes** ^a^	**No**	**OR** ^b^	**95% CI**
Physical health	22.1	12.2	2.03	1.43, 2.87
Mental health	12.2	5.4	2.42	1.55, 3.76
Inactivity health	28.2	16.9	1.93	1.23, 3.02
General health	22.7	15.9	1.56	1.15, 2.10
Unhealthy days	26.7	15.9	1.93	1.40, 2.68
**Physically inactive**				
**Female **	**Yes** ^a^	**No**	**OR** ^b^	**95% CI**
Physical health	26.8	10.6	3.08	2.28, 4.15
Mental health	17.2	8.4	2.27	1.59, 3.23
Inactivity health	25.4	12.4	2.40	1.63, 3.53
General health	28.0	13.2	2.55	1.93, 3.38
Unhealthy days	34.0	16.1	2.67	2.05, 3.49

Note: ^a^ indicates physical inactivity status. ^b^ is the odds of poor health for those who are physically inactive compared to those not physically inactive. Each overall model showed a significant (*P* < 0.05) sex-by-PIA interaction.

**Table 3:** Odds of poor HRQOL for physically inactive rural adults compared to non-physically inactive rural adults, 2013

**Male **	**OR** ^a^	**95% CI**	**OR** ^b^	**95% CI**
Physical health	1.99	1.41, 2.83	1.79	1.22, 2.63
Mental health	2.45	1.58, 3.82	2.10	1.28, 3.44
Inactivity health	1.86	1.19, 2.90	1.92	1.19, 3.10
General health	1.51	1.11, 2.04	1.41	1.01, 1.99
Unhealthy days	1.91	1.39, 2.64	1.69	1.18, 2.42
**Female **	**OR** ^a^	**95% CI**	**OR** ^b^	**95% CI**
Physical health	2.84	2.12, 3.81	2.58	1.86, 3.59
Mental health	2.48	1.73, 3.55	2.16	1.46, 3.21
Inactivity health	2.37	1.61, 3.47	2.00	1.31, 3.05
General health	2.36	1.79, 3.10	1.80	1.32, 2.47
Unhealthy days	2.56	1.99, 3.39	2.43	1.81, 3.26

Note:^a^ indicates odds ratios (OR) are age adjusted. ^b^ indicates ORs are adjusted for age, race, and income. Fully-adjusted interactions were significant (*P* < 0.05) for physical health, general health, and unhealthy days. Overall combined mental health OR: 2.12; 95% CI: 1.56-2.89. Overall combined inactivity health OR: 1.95; 95% CI: 1.43-2.67

## Discussion

The primary purpose of this study was to investigate the relationship between PIA and HRQOL in a rural adult population. This relationship was clearly established by evidence showing that rural adults reporting no PA were more likely to report poor HRQOL in crude, age-, and fully-adjusted models. Moreover, this relationship persisted across all five measures of HRQOL (general health, physical health, mental health, and inactivity health, and unhealthy days).

A secondary purpose of this study was to examine the extent to which sex differences exist in the PIA and HRQOL relationship among rural adults. Results from this focus of the study showed that among rural adults who were physically inactive, females were generally more likely to report poor HRQOL as compared to their male counterparts. These sex differences were consistently seen across all five HRQOL measures in the crude analyses. However, in the adjusted analyses, mental health and inactivity health lost their significant sex differences. This implies that rural males and females who are physically inactive experience similar mental health and experience a similar level of inactivity health.

A final result of this study was seen when investigating poor HRQOL (general health) prevalence among physically inactive rural adults across BMI categories. Sex differences among physically inactive rural adults were apparent in this analysis, where the prevalence of poor HRQOL followed a significant direct linear relationship with BMI category. That is, as BMI category moved from lower BMI (underweight) to higher BMI (obese), the prevalence of poor HRQOL also increased significantly in rural males. Unexpectedly, the opposite was seen in physically inactive rural females. That is, as BMI category moved from lower BMI (underweight) to higher BMI (obese), the prevalence of poor HRQOL decreased. Although this trend among physically inactive rural females was not significant, it was however suggestive (*P *< 0.10). This sex difference implies that overweight and obese conditions has a different HRQOL affect on physically inactive rural females than it does on physically inactive rural males. A possible explanation for this sex difference is that physically inactive rural males who are underweight or normal weight are able to perceive their health status as adequate, despite being physically inactive. Whereas physically inactive rural females who are underweight or normal weight display perceived health status more closely tied to their physical activity behavior. However, further research is needed to explain these findings.

One limitation of this study is its cross-sectional nature which limits a study’s findings to correlational inferences as opposed to cause-and-effect generalizations. However, the findings in this study are consistent with those found in randomized controlled trials. For example, Swedish researchers recently reported improvements in HRQOL among intervention participants who were prescribed physical activity as part of a randomized controlled trial.^21^ Another limitation of this study is its data collection methodology, with data collected via telephone. It is commonly understood by survey researchers that certain segments of the population, such as the indigent or minorities, may not have access to a telephone. These subpopulations may also be less likely to be physically active and more likely to exhibit poor HRQOL. However, there is reason to speculate that more respondents from such segments may only increase the strength of our reported relationship. A final limitation of this study is the use of the self-reported assessments of PIA, HRQOL, and BMI. Although these limitations are well noted, it stands to reason that there is less measurement error in the assessment of PIA than in the assessment of PA. There is also reason to believe that the use of five different measures of HRQOL would allow for an averaging effect of measurement error. Finally, self-reported BMI has been shown to be relatively valid and reliable in telephone surveys.^22^

This study has many strengths worth mentioning. First, data for this study are from a large representative sample of rural adults 18+ years of age from a Western region US state. The complex multi-stage sampling employed in this survey safeguards representation from all subgroups normally left out of non-probability samples. Therefore, these data allow for much more confident generalizations concerning rural adults and their health status. A second strength of this study is its use of five different measures of HRQOL (general health, physical health, mental health, and inactivity health, and unhealthy days). Using five different HRQOL outcomes measures, and finding similar relationships across each, is testament to the robustness of these study findings. A final strength worth mentioning is the use of multivariate logistic regression models to examine the relationship between PIA and HRQOL. These models included commonly recognized confounding demographic variables that otherwise could distort study inferences.

## Conclusion

Results from this study show that PIA is a strong predictor of poor HRQOL even after controlling for confounding variables. Furthermore, physically inactive rural women were significantly more likely to report poor levels of HRQOL than physically inactive rural men. BMI category may be a useful predictor of HRQOL in physically inactive rural adults, however, it predicts differently for males than for females. Health promotion interventions aimed at rural adults should plan component strategies differently for males and females. Specifically, there is more opportunity to increase the HRQOL in rural physically inactive females, than for males, by improving their physical activity behavior.

## Acknowledgments

The author would like to thank the US Centers for Disease Control and Prevention (CDC) for use of the BRFSS dataset.

## Ethical approval

This study used public domain data as a secondary data analysis. Therefore, institutional review board approval was not required.

## Competing interests

The author declares that he has no competing interest.

## Author’s contribution

PDH designed, managed, and analyzed this study as well as wrote this paper.
